# Dye- and fluorescence-based assay to characterize symplastic and apoplastic trafficking in soybean (*Glycime max* L.) endosperm

**DOI:** 10.1186/s40529-019-0271-0

**Published:** 2019-09-23

**Authors:** Ming-Der Shih, Jian-Shin Lin, Mei-Jane Fang, Yuan-Ching Tsai, Yue-Ie C. Hsing

**Affiliations:** 1 Institute of Plant and Microbial Biology, Academia Sinica, 128, Sec. 2, Academia Rd, Nangang, Taipei, Taiwan; 2 Department of Agronomy, National Chiayi University, Chiayi, Taiwan; 3 Department of Agronomy, National Taiwan University, Taipei, Taiwan

**Keywords:** Cotyledon, Endosperm, Movement, Size exclusion limit, Soybean, Symplastic tracer

## Abstract

**Background:**

Endosperm is a triploid tissue in seed resulting from a sperm nucleus fused with the binucleate central cell after double fertilization. Endosperm may be involved in metabolite production, solute transport, nutrient storage, and germination. In the legume family (Fabaceae), with the greatest number of domesticated crops, approximately 60% of genera contain well-differentiated endosperm in mature seeds. Soybean seeds, the most important legume crop in the worlds, have endosperm surrounding embryos during all stages of seed development. However, the function of soybean endosperm is still unknown.

**Results:**

Flow cytometry assay confirmed that soybean endosperm was triploid. Cytobiological observation showed that soybean endosperm cells were alive with zigzag-shape cell wall. Soybean endosperm cells allowed fusion proteins (42 kDa) to move from bombarded cells to adjacent unbombarded-cells. Such movement is not simple diffusion because the fusion proteins failed to move into dead cells. We used symplastic tracers to test the transport potential of soybean endosperm. Small organic dye and low-molecular-weight symplastic tracers revealed fast symplastic transport. After a treatment of an inhibitor of ATPase, *N*,*N*′-dicyclohexylcarbodiimide (DCCD), symplastic transport was blocked, but all tracers still showed fast apolopastic transport. The transport speed of 8-hydroxypyrene-1,3,6-trisulfonic acid in endosperm was 1.5 to 3 times faster than in cotyledon cells or Arabidopsis embryos.

**Conclusions:**

Soybean endosperm is a membrane-like, semi-transparent, and fully active tissue located between the seed coat and cotyledon. Soybean endosperm cells allowed macromolecules to move fast via plasmodesmata transport. The size exclusion limit is larger for soybean endosperm cells than its cotyledon or even Arabidopsis embryo cells. Soybean endosperm may be involved in fast and horizontal transport during the mid-developmental stage of seeds.

## Background

Endosperm is the tissue that is formed from double fertilization in flowering plant seeds, the process whereby a sperm nucleus fuses with the binucleate central cell. Seeds of approximately 70% flowering plants have endosperm cells (Brown and Lemmon 2007), most of them are triploidy. However, several plant families, such as Oenograceae, Cabombaceae, and Illiciaceae, form diploid endosperm originating from a central cell with only one polar nucleus (Williams and Friedman 2002). Depending on the species, endosperm can be (1) absent (e.g., Podostemonaceae), (2) short-lived (e.g., Orchidaceae), (3) absorbed by the developing embryo, such as Arabidopsis, pea (*Pisum sativum*), and mongo (*Mangifera indica*), or (4) maintained in mature seeds, such as cereals, *Medicago truncatula*, and soybean (see review in Baroux et al. 2002). Seeds with fully differentiated endosperm in the maturation stage are called albuminous. In dicot albuminous seeds, endosperm may be involved in metabolite production, solute transport, and accumulation in the embryo (Melkus et al. 2009). In monocot albuminous seeds, including all cereals, endosperm usually represents a large amount of the seed volume and plays important roles in nutrient storage and germination (Baroux et al. 2002; Brown and Lemmon 2007).

The legume family (Fabaceae) has the greatest number of domesticated crops from 41 species (Harlan 1992). Because such seeds provide high-quality protein and oils for human food or livestock feed, the seed structure and function are important issues in agriculture. Generally, legume seeds have relatively uniform structures, such as seed coat, embryo, and cotyledons. However, the presence, amount, and functions of endosperm in mature legume seeds are diverse. In some species, including several important crops such as pea, faba bean (*Vicia faba*), chickpea (*Cicer arietinum*), common bean (*Phaseolus vulgaris*), and cowpea (*Vigna unguiculata*), endosperm forms at the early developmental stage but is fully absorbed and obliterated during embryo enlargement and is characteristically absent in mature seeds (Yeung and Cavey 1988; Goldberg et al. 1994). In contrast to species with absent endosperm, more than 450 genera in the legume family maintain well-developed endosperm in mature seeds (Kirkbridge et al. 2003), with the proportion of endosperm representing from 1 to 60% of the seed size (Anderson 1949). For some genera such as fenugreek (*Trigonella foenum*–*graecum*), crimson clover (*Trifolium incarnatum*) and lucerne (*Medicago sativa*), endosperm has a thick structure and functions as storage tissue (Reid and Meier 1972; McClendon et al. 1976; Reid and Bewley 1979). In some other genera, such as soybean, *M. truncatula*, and *Trifolium repens*, endosperm tissues remain as a continuous thin layer of living cells and surround the embryo. The structures of legume endosperm are diverse, and part of their functions are still unknown (Thorne 1981; Jakobsen et al. 1994; Ma et al. 2004; Bolingue et al. 2010).

Because of no direct connection between maternal seed coat and fatal embryo (Thorne 1981), post-phloem transport is very important in solute movement inside the seed. To understand the transport pathway, organic or fluorescent tracers provide an in vivo way to observe transport pathways of tissues at a microscopic scale (Barnabas 1994). Methylene blue is commonly used to stain the nucleus and cytoplasm of eukaryotic cells, and the staining could be prevented by inhibiting ATPase (Kiernan 1974). In plant cells, methylene blue has also been used to stain the cell wall and show long-distance movement via plasmodesmata transport (Scarth 1926; Wolk 1973). A small symplastic tracer, 8-hydroxypyrene-1,3,6-trisulfonic acid (HPTS, 0.5 kDa), has the characteristics of stability, non-toxicity, and membrane-impermeable (Zhujun and Seitz 1984). Fluorescein isothiocyanate (FITC)-dextran is used as a fluorescent high-molecular-weight probe to study cell processes such as cell permeability, endocytosis, and biomolecular delivery (Cole et al. 1990). Previous work also suggested that ATPase inhibitors, such as *N*,*N*′-dicyclohexylcarbodiimide (DCCD), prevented the uptake of FITC-dextran in yeast (*Saccharomyces cerevisiae*) and animal cells (Makarow and Nevalainen 1987; Fuchs et al. 1989; Anbari et al. 1994). DCCD had been demonstrated to prevent NTP hydrolysis and inhibited delta pH formation (Ugurbil et al. 1978). The possible mechanism might be that DCCD changed the conformation of ATPase and thus impaired the function of catalytic sites (Penefsky 1985).

Previous studies had been demonstrated that several proteins performed the cell-to-cell movement via plasmodesmata (Noueiry et al. 1994). To clearly trace protein movement, GFP and its fusion proteins had been used for plasmodesmata-mediated symplastic transport analysis. Those results suggested that plasmodesmata had size exclusion limit (SEL) depending on the cell types (Lucas 1999). For example, only 27-kDa GFP but not larger GFP fusion proteins (37 to 67 kDa) moved symplastically among *Arabidopsis* root tip cells (Meyer et al. 2004; Hoth et al. 2005; Stadler et al. 2005a, b). Phloem sap of pumpkin (*Cucurbita maxima*) allowed 20–40 kDa proteins transport via plasmodesmata (Balachandran et al. 1997; Lucas 1999). Hence, SEL of plasmodesmata-mediated protein movement was below 40 kDa (Stadler et al. 2005a; Yadav et al. 2014). *GmPM16* is a soybean *LEA 4* gene encoding a basic and small hydrophilic LEA protein. We previously used this protein for several cytological, biological, and structural studies (Shih et al. 2004, 2008, 2012). *GmPM16* gene is highly expressed in maturing seeds but is not responsive to abiotic stress treatment in vegetative tissues (Shih et al. 2004). The encoded proteins of *GmPM16* gene might play the roles of molecular chaperone during drying (Shih et al. 2004, 2008).

Soybean is one of important legume crops in the world. More than 300 million tons per year of soybean seeds are produced for food and industry (FAOSTAT, http://www.fao.org/faostat/). Although soybean seeds are important in agriculture and industry, there is little research focusing on its endosperm cells. In the current study, we examined soybean endosperm and found that it is a membrane-like tissue and surrounds the embryonic structures. Endosperm is observed as a single layer of living cells located between seed coat and cotyledon. These results led us to investigate the transport ability of soybean endosperm. To test the ability, we used GmPM16 fusion protein (42 kDa) and several fluorescent symplastic tracers (0.5 to 70 kDa) to measure the transport limits of endosperm cells. We reveal that soybean endosperm may facilitate large protein and fluorescent dye molecule movement among cells. The organic and small fluorescent dyes showed fast cell-to-cell movement. Therefore, our results suggest that soybean endosperm may mediate solution transport in soybean, with fast horizontal symplastic and apoplastic movement.

## Methods

### Plant materials

Seeds of the soybean variety Shi-shi were kindly provided by the Kaohsiung Agricultural Research and Extension Station (Pingtung, Taiwan). Plants were grown under field conditions at the Academia Sinica campus with natural light and photoperiod. Seeds were harvested at mid-development stage, 35 days after flowering (DAF).

### Ploidy estimation

Ploidy of soybean endosperm was estimated by as described by Dolezel and Bartos (2005) with modification. Briefly, nuclei of endosperm and leaf were released into a nuclei isolation buffer (50 mM glucose, 15 mM KCl, 15 mM NaCl, 5 mM EDTA, 50 mM sodium citrate, 0.5% (v/v) Tween 20, 50 mm HEPES, 0.5% (v/v) β-mercaptoethanol, pH 7.2) by mechanical homogenization before use. Extracted nuclei were was stained with CyStain PI absolute P (Partec, Germany) before analysis by flow cytometry (MoFlo XDP Cell Sorter, Beckman, USA) with laser excitation at 357 nm.

### Cryo-scanning electron microscopy (cryo-SEM)

Cryo-SEM observation was as described by Thorne (1981) with modification. Fresh developing soybean seeds were rapidly frozen in liquid nitrogen and were coated with gold in a vacuum (0.2 kPa, 3 min, ionization current 2 mA) before observation. The sample was analyzed by Philip FEI Quanta 200 SEM (The Netherlands) with the cryo system Quorum PP2000TR FEI at an accelerating voltage of 10 kV and a working distance of 15 mm and temperature < − 130 °C.

### Confocal microscopy

Confocal microscopic observation was as described by Musielak et al. (2015) with modification. A 200-μg amount of propidium iodide (PI) (Sigma-Aldrich, USA) was freshly dissolved in 10 ml phosphate buffered saline before use. Endosperm tissues were soaked in PI solution, and images were obtained within 30 min under a Zeiss 510 meta laser-scanning confocal microscope (Carl Zeiss MicroImaging GmbH, Jena, Germany) with 910/0.45 air or 963/1.2 water immersion objectives. The emission spectra were recorded between 370 and 650 nm by using a Jasco 6500 Fluorometer with excitation wavelength 350 nm.

### Plasmid construction

Cloning was performed essentially as described (Sambrook and Russell 2001). The modified plasmid pCass vector (Shi et al. 1997) containing an enhanced green fluorescent protein (GFP) under control of the CaMV35S promoter was used for bombardment assay. The SmaI fragment of GmPM16 (Shih et al. 2004) containing the coding region was amplified by PCR, then cloned into the plasmid pCass vector. The coding sequence of the clone was fused in-frame to the 5′ end of the enhanced GFP gene and termed pCass-GmPM16. The fusion plasmids were isolated by use of a plasmid Miniprep kit (Qiagen, Germany).

### Bombardment assay

For protein movement assay, 10 to 20 pieces of endosperm were peeled off and pre-cultured for 1 day on 1/2 Murashige and Skoog (MS) basal medium supplemented with 3% (m/v) sucrose and 0.8% (m/v) agar at room temperature under darkness. Particle bombardment involved use of a Biolistic Particle Delivery System (Bio-Rad) PDS-1000. Before bombardment, 5 or 0.5 μg plasmid DNA was coated onto gold particles with diameter 1.6 μm (INBIO GOLD, Australia), which were then bombarded into soybean endosperm layers by acceleration at 1100 psi in a chamber vacuum pressure of 27 mmHg. The distance between the projectile source and the sample was 6 cm. Bombarded endosperm was peeled off and placed in 1/2 MS basal medium solution supplemented with 3% (m/v) sucrose for 16 h. GFP in all experiments was excited at 488 nm, with emission at 505–550 nm, and a transmitted light image was collected for a reference. Images were obtained under a Zeiss 510 meta laser scanning confocal microscope (Carl Zeiss MicroImaging GmbH, Jena, Germany) with 910/0.45 air or 963/1.2 water immersion objectives.

### *N*,*N*′-Dicyclohexylcarbodiimide (DCCD) preparation

DCCD (Sigma, USA) was diluted with 100% ethanol. The final concentration of DCCD was 0.1 mM in any treatment. DCCD solution was prepared before use.

### Organic dye staining

An organic dye, methylene blue (320 Da, Sigma-Aldrich, USA), was prepared in distilled H_2_O at 10 mg/l for testing transport efficiency of endosperm or cotyledon epidermis. A 1-μl amount of dye solution was applied onto endosperm or cotyledon epidermis, then observed after 20 min. For ATPase inhibitor assay, 1 μl dye and DCCD (0.1 mM) mixture was applied onto the surface of endosperm or cotyledon epidermis, then observed after 20 min by optical stereomicroscopy (Zeiss Lumar V12, Carl Zeiss MicroImaging GmbH, Germany). Stained area was estimated by using ImageJ (http://imagej.nih.gov/ij/). The increasing ratio was calculated as stained area at 20 min (cm^2^)/stained area at 0 min (cm^2^). Statistical analysis involved using SPSS v20 (SPSS, USA) with statistical significance set at *p* < 0.05.

### Transport of fluorescent tracers

8-Hydroxypyrene-1,3,6-trisulfonic acid (HPTS; 524 Da, Molecular Probes, USA) and fluorescein isothiocyanate-conjugated dextran (FITC-dextran) at 10, 20, 40, and 70 kDa (Sigma-Aldrich, USA) were prepared in 1/2 MS medium with 0.8% agar to 5 mg/ml. For uptake assay, soybean seeds were cut into 2 parts after removed the seed coat and placed on solid MS medium with different fluorescent tracers for 20 min (for HPTS) or 24 h (for FITC-dextran). For estimating cell-to-cell transport speed, soybean seeds with the seed coat removed or both seed coat and endosperm removed were cut into 2 parts and placed on solid MS medium with HPTS or 10-kDa FITC-dextran for 3 min. The movement distance was measured every 30 s. All images were taken under a fluorescence stereomicroscope (Zeiss Lumar V12) or Zeiss 510 meta laser scanning confocal microscope (Carl Zeiss MicroImaging GmbH, Germany) with excitation at 405 nm and emission at 505–525 nm to trace the route of fluorescent probes.

## Results

### General structures of soybean endosperm

**Fig. 1:**
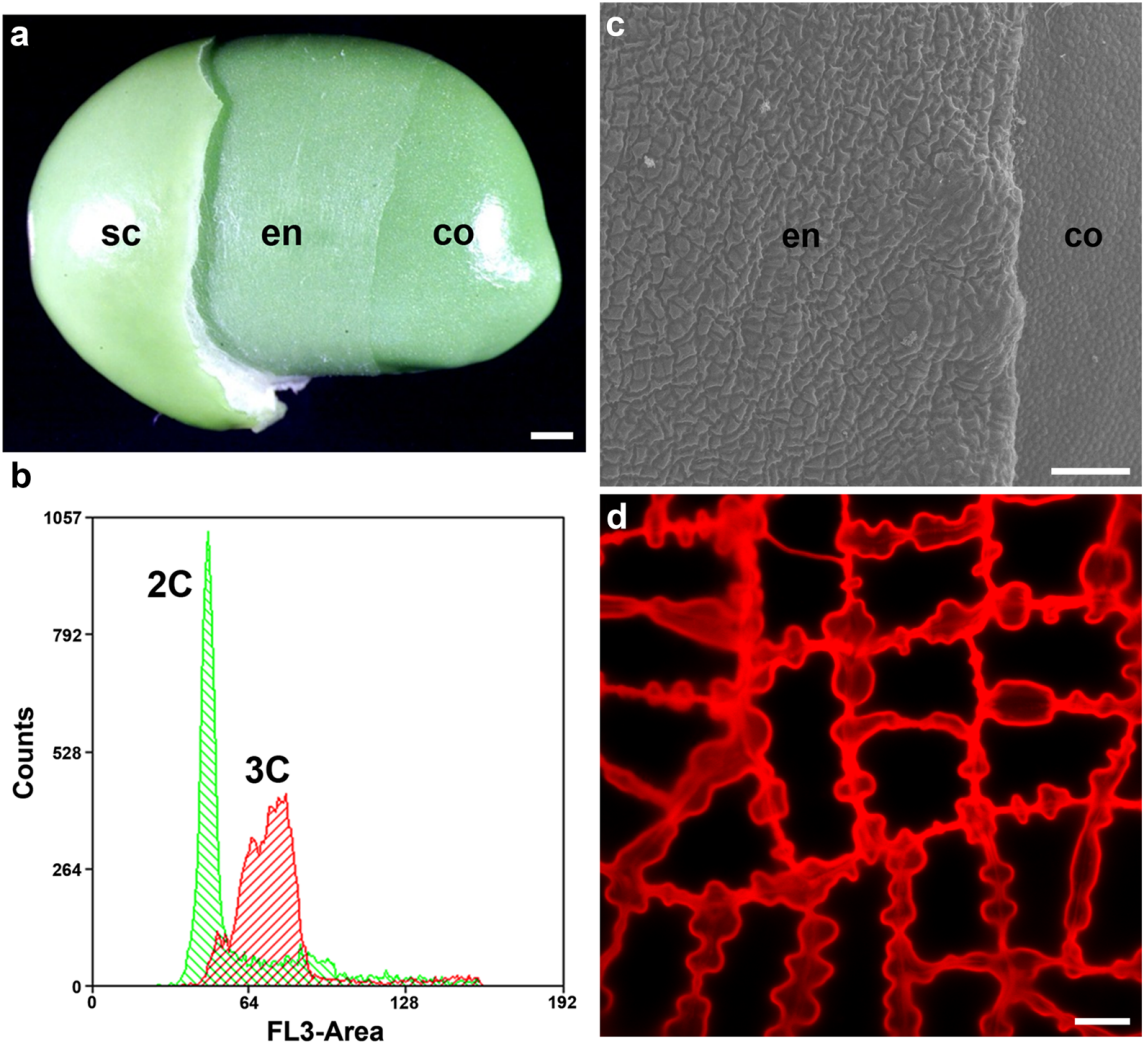
The structural morphology and ploidy of soybean endosperm. **a** Photographs of soybean seed. Parts of seed coat (sc) and endosperm (en) were removed. Endosperm is an independent and membrane-like tissue, located between the seed coat and cotyledon (co). **b** Flow cytometry of fresh leaves (green) from mature plants, and peeled endosperm (red) from soybean seed at 35 days after flowering (DAF). The diploid internal reference produced two peaks (green area), including a major peak with ×2DNA quantity and a minor peak corresponding to ×4 DNA. The peak of the red area corresponds to ×3 DNA. **c** Cryo-scanning electron microscopy (cryo-SEM) of soybean endosperm and cotyledon epidermis cells at 35 DAF. Cell wall ingrowth of soybean endosperm is identified in endosperm. **d** Fluorescence microscopy of propidium iodide-stained cell wall from soybean endosperm. sc, seed coat; en, endosperm; co, cotyledon. Scale bars = 1 mm for **a**, 100 μm for **c** and 10 μm for **d**

After carefully removing the seed coat, we observed a thin layer between the seed coat and cotyledon (Fig. 1a). Flow cytometry revealed that this tissue was triploid endosperm (Fig. 1b). The peeled endosperm tissue in 35-DAF seed was further revealed by cryo-SEM (Fig. 1c; left side, endosperm cells; right side, epidermal layer of cotyledon) and optical microscopy (Fig. 1d). The endosperm cells were larger than cotyledon epidermal cells, had a zigzag shape, and varied in size. Cotyledon epidermal cells were small, ovary-shaped, and were similar in size (Fig. 1c). The zoom-in view of the soybean endosperm cell revealed a striking thin or thick cell wall structures (Fig. 1d).

### Protein movement with active transport in endosperm cells

**Fig. 2:**
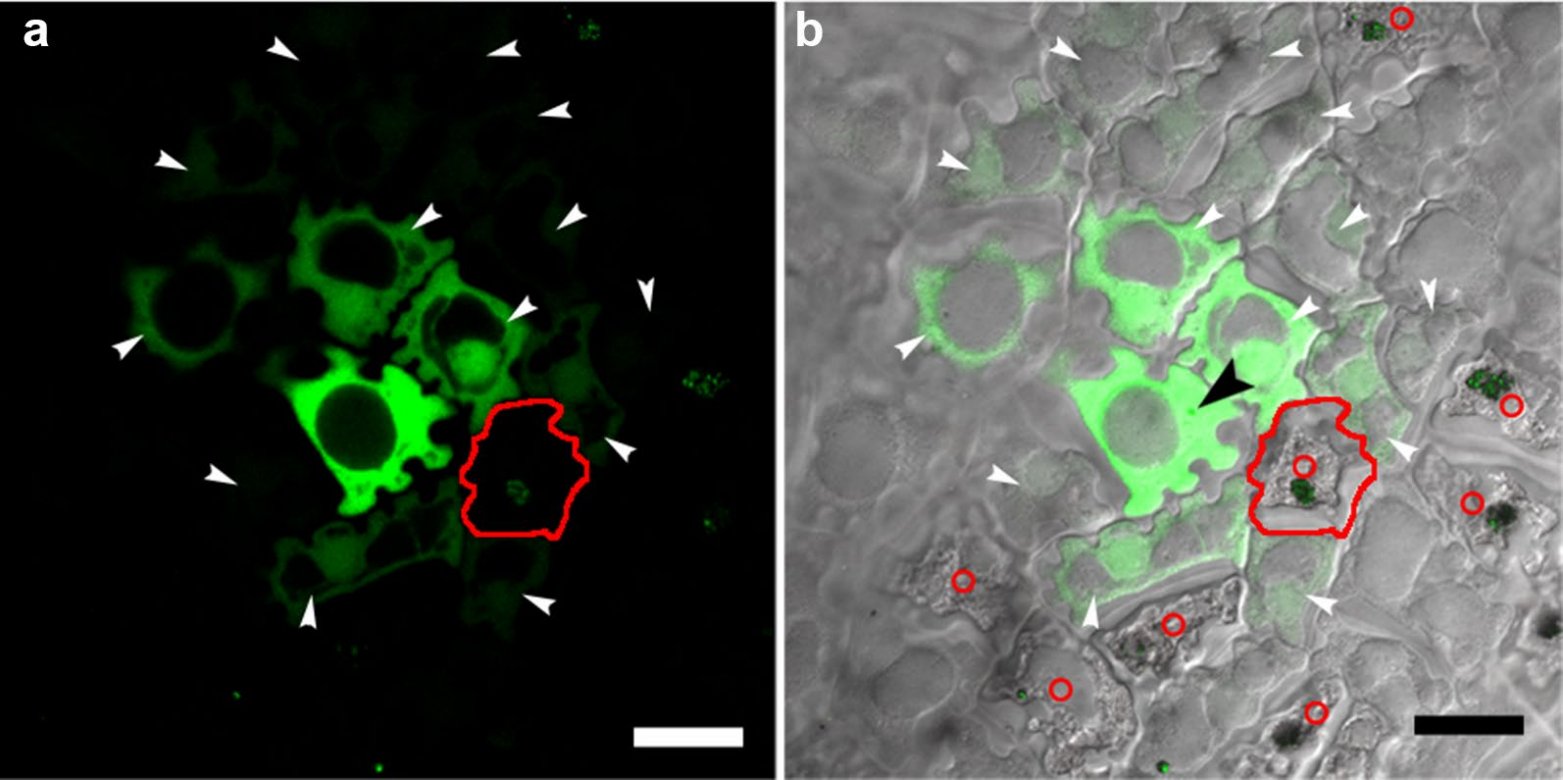
Movement of GmPM16-GFP fusion proteins in soybean endosperm. The cells were imaged by **a** epi-fluorescence and **b** overlapping fluorescence and phase contrast. The positions of gold particles in nuclei are marked by a black arrowhead in **b**. The fluorescent cells caused by fusion proteins movement are marked by white arrowheads. The red circles indicate the destroyed cells containing gold particles. Scale bars = 20 μm

Previous work suggested that SEL of most cells was below 40 kDa (e.g. Balachandran et al. 1997; Lucas 1999; Stadler et al. 2005a; Yadav et al. 2014). The molecular weight of GmPM16-GFP fusion proteins was 42 kDa, and thus the fusion protein was used to reveal the SEL of soybean endosperm. Figure 2 illustrates the movement of GmPM16-GFP fusion proteins. The fusion protein was synthesized in the cell harboring a bombarded particle (indicated by a black arrowhead; Fig. 2b), then moved to more than 10 adjacent cells (Fig. 2a). However, no fluorescence signal was detected in 7 nearby cells with plasmolysis (indicated by red circles). The destroyed cell circled by a red line located right next to the fluorescent cell with gold particles and had no fluorescence. Four living cells surrounding the circled one are fluorescent (Fig. 2b). Therefore, the GFP fusion proteins failed to move into dead cells, and instead bypassed them, which indicates that the protein molecules may move freely in living cells but not dead ones.

### ATP inhibitor DCCD blocks dye transfer on endosperm

**Fig. 3:**
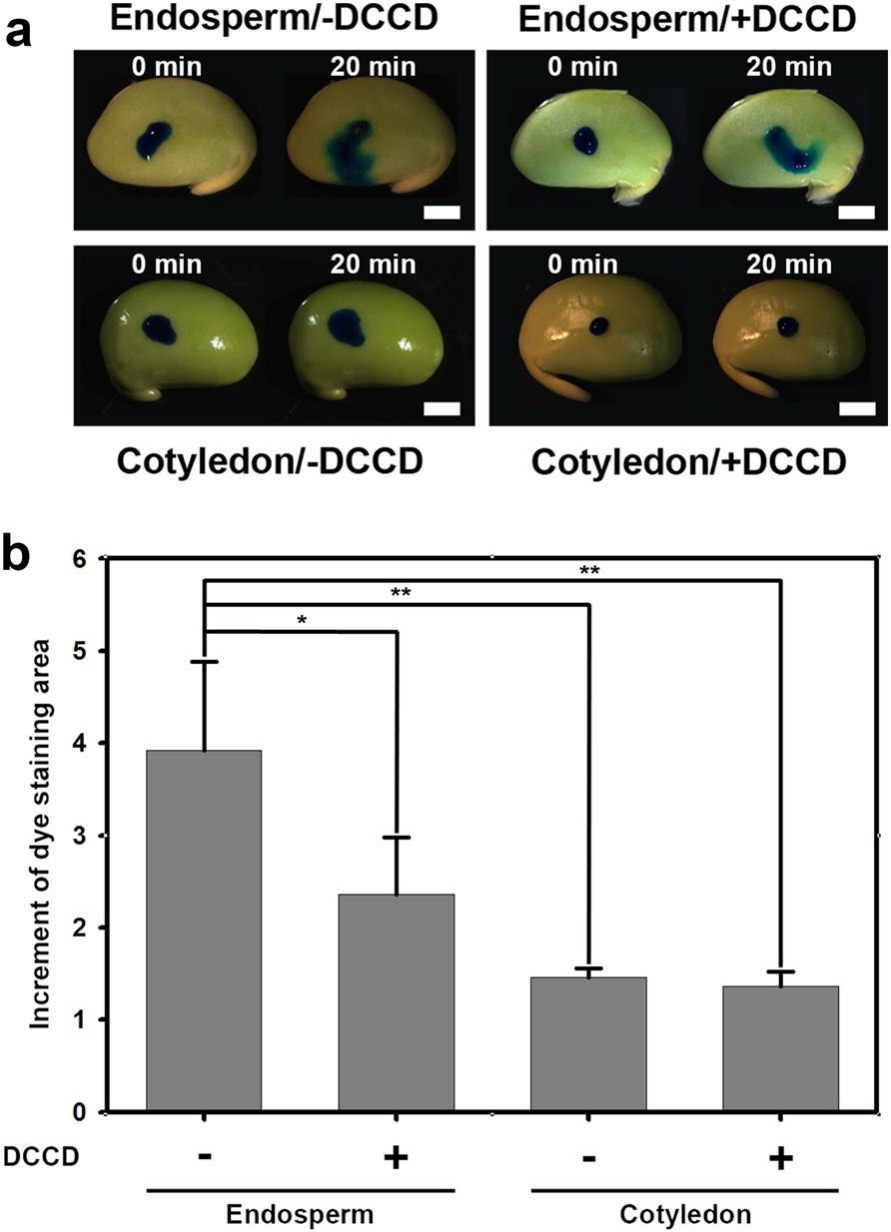
Methylene blue staining on endosperm or cotyledon surface. **a** Increment of dye staining-area at 0 and 20 min after application of methylene blue on soybean endosperm or cotyledon, with or without DCCD treatments. **b** The dye staining area of endosperm without DCCD treatment was significantly larger than in endosperm with DCCD treatment and cotyledon with or without DCCD treatments. The data were analyzed by ANOVA with Scheffe’s S method (*p < 0.05; **p < 0.01). Data are mean ± SE

To test the transport potential of soybean endosperm, we applied 1 µl methylene blue solution onto the endosperm or cotyledon surface with or without DCCD treatment for 1 h. The dye stained areas were measured every 5 min for 20 min (Fig. 3a). For endosperm, the stained area of methylene blue increased approximately fourfold from 0 to 20 min (increment of staining area: 3.92 ± 0.83-folds, n = 5), whereas with DCCD treatment, the stained area increased 2.4-fold from 0 to 20 min (increment of staining area: 2.36 ± 0.62 folds, n = 5). By contrast, the staining areas on cotyledons with or without DCCD treatment were about 1.4-fold higher at 20 min than at 0 min (increment of staining area: 1.46 ± 0.10 folds, n = 5, and 1.36 ± 0.16-folds, n = 5, respectively). The staining area of endosperm without DCCD treatment were significantly larger than on endosperm with DCCD treatment (*p* < 0.05) and on cotyledon with or without DCCD treatments (*p* < 0.01) (Fig. 3b). These results indicate that DCCD significantly blocked the dye transfer on endosperm, and soybean cotyledon has poor efficiency of dye transfer. Thus, it suggests that the active transport in the endosperm cells is more important than that in the cotyledon.

### Transport pathways of soybean endosperm

**Fig. 4:**
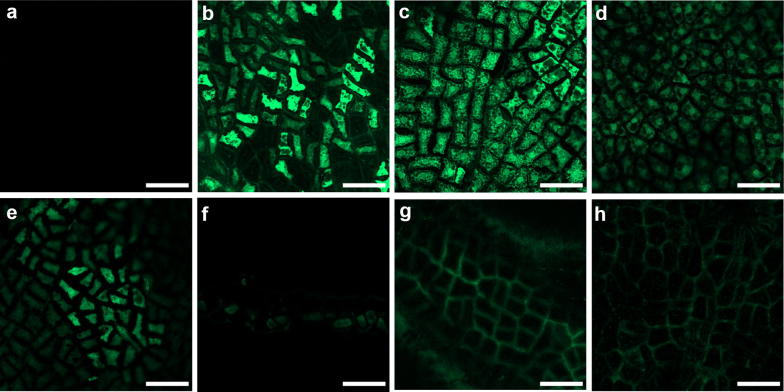
Cell-to-cell movement of fluorescent symplastic tracer in soybean endosperm. Isolated soybean endosperm from 35 DAF seeds loaded with **a** 1/2 MS media, **b** HPTS; **c** 10-kDa, **d** 20-kDa, **e** 40-kDa, or **f** 70-kDa F FITC-dextran; or **g** 10-kDa or **h** 70-kDa FITC-dextran with DCCD. Scale bars = 50 μm

We used two different fluorescent symplastic tracers, a small HPTS (0.5 kDa) and a series of FITC-dextran with various molecular weights (10, 20, 40, or 70 kDa), to trace the cell-to-cell movement ability of endosperm (Fig. 4). Endosperm was peeled from soybean seed, then soaked in HPTS solution for 5 min or in FITC-dextran solutions for 24 h. HPTS as well as 10-, 20-, and 40-kDa FITC-dextran moved freely among endosperm cells (Fig. 4b–e). By contrast, 70-kDa FITC-dextran stayed at the cut edge of endosperm and had no further movement (Fig. 4f). After DCCD treatment, fluorescent signals were identified only at the cell wall region but not in endosperm cells (Fig. 4g, h). No spontaneous fluorescence was detected under the same excitation/emission condition (Fig. 4a).

### Transport speed of soybean endosperm

**Fig. 5:**
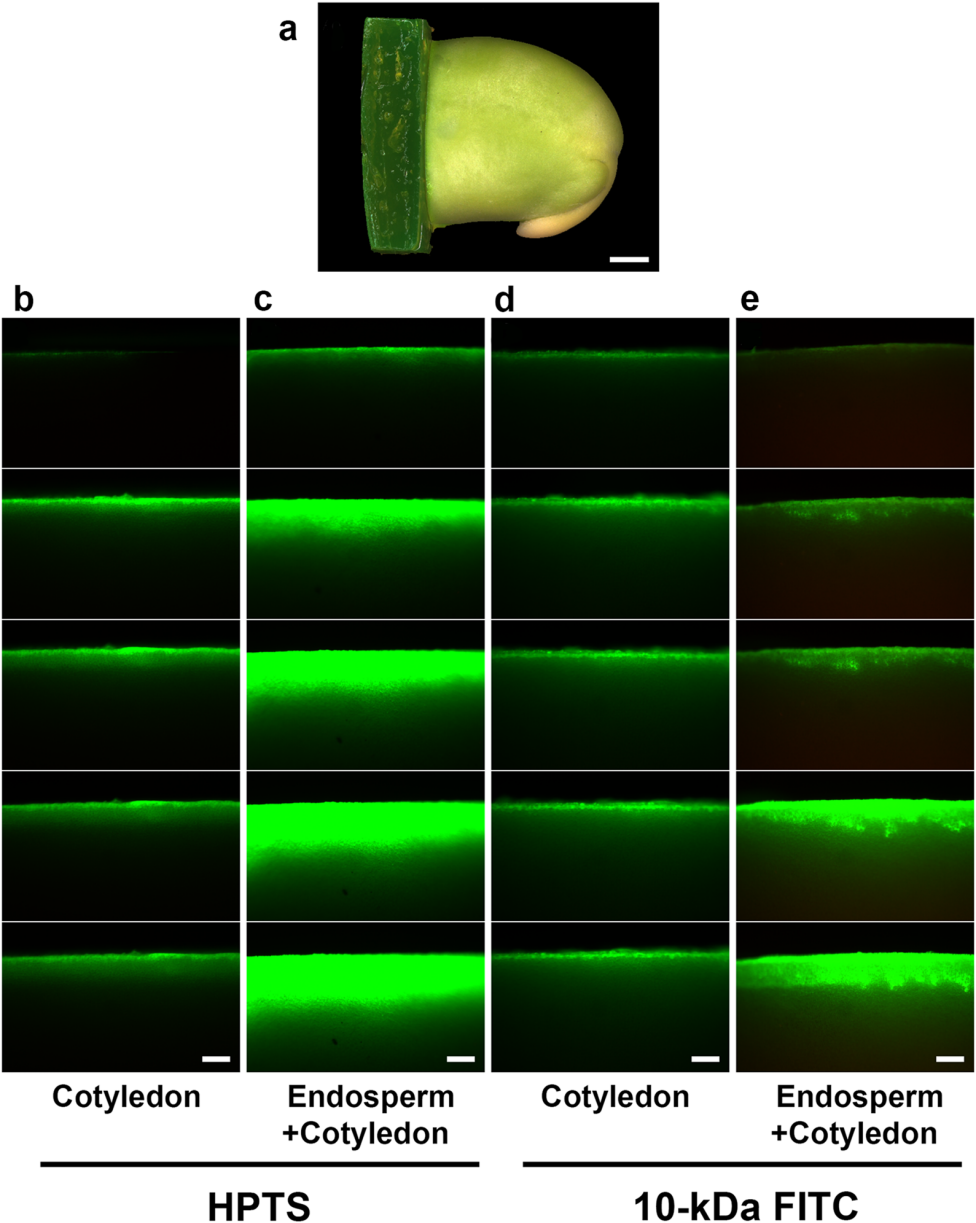
Movement of HTPS and 10-kDa FITC-dextran in endosperm or cotyledon tissue. **a** Soybean seed was cut at the opposite site of the embryonic axis. The cut edge was contacted with agar gel with symplastic tracer. The tracer transferred to cells starting from the cut end of **b** cotyledon only or **c** endosperm on cotyledon with HTPS or **d** cotyledon only or **e** endosperm on cotyledon with 10-kDa FITC-dextran at 0, 0.5, 1, 2, and 3 min (from upper to button). Scale bars = 200 μm

Because the tracer movement occurred initially from broken cells at the cut edges and followed a certain direction, the cut edges provided a baseline for measuring endosperm transport speed. Seeds with the seed coat peeled or with both the seed coat and endosperm peeled were cut, then immediately placed on gels containing HPTS or 10-kDa FITC-dextran (Fig. 5a). The tracer movements were measured every 30 s. In endosperm, HPTS and 10-kDa FITC-dextran moved 0.6 and 0.4 mm, respectively, in 3 min (Fig. 5c, e). However, in cotyledon, HPTS moved 0.2 mm but 10-kDa FITC-dextran was blocked at the cut edge (Fig. 5b, d). From similar experiments in Arabidopsis embryos (Kim et al. 2002), HPTS moved approximately 0.2 mm from the base to the top in 3 min.

## Discussion

We confirm that the membrane-like and semi-transparent tissue at mid-developmental stage of soybean seed is a layer of endosperm cells which are triploid and alive, with zigzag shaped cell wall (Fig. 1). Our results also show that soybean endosperm cells allowed macromolecules (up to 40 kDa) to move into adjacent cells via plasmodesmata (Fig. 2). In addition, soybean endosperm showed fast symplastic transport as well as apoplastic transport after ATPase inhibitor treatment (Figs. 3, 4). The speed of a low-molecular-weight symplastic tracer moving in endosperm cells was faster by 1.5- to 3-fold than in Arabidopsis embryos (Kim et al. 2002) or soybean cotyledon epidermis cells (Fig. 5).

At mid-developmental stage, soybean seeds begin a period of rapid seed growth. During this stage, endosperm covers whole seed and has exuberant vitality (Miller et al. 1999; Shih et al. 2010) and may have a biological role. A series of morphological observations of developing seeds demonstrated the existence of endosperm tissue in other modern soybean varieties, including those from America or Japan, and in wild-related species, such as *G. soja*, *G. tomentella*, and *G. tabacina* (Hsieh et al. 2001). Hence, soybean endosperm is common in morphological and structural features in the genus *Glycine*. We also described that endosperm is easily peeled from maturing soybean seed, thus this is the first case to use isolated soybean endosperm for transient assay. With the identified macromolecule movement, we considered a possible transport role in soybean endosperm. Although some cells were destroyed by gold particles located near the most fluorescent cell, GFP fusion proteins could bypass shrinking cells to other surrounding cells. Cytosolic flow in endosperm cells may not be simply passive diffusion but also energy-dependent symplastic transport.

The symplastic transport is the solute transport pathway mediated by plasmodesmata. The ability of plasmodesmata may be associated with their size exclusion. The upper limit of plasmodesmata in terms of size of macromolecules allows for easy transport among cells (Tucker 1982; Goodwin 1983; Kempers et al. 1993; Kempers and Van Bel 1997). The average SEL of plant cells measured by protein movement assay was usually below 40 kDa in previous studies using bombardment of GFP fusion genes (Meyer et al. 2004; Hoth et al. 2005; Stadler et al. 2005a, b; Yadav et al. 2014). An embryo-defective Arabidopsis mutant, *increased size exclusion limits 1* and *2* (*ise1* and *2*), showed changed SEL during the shift from heart-shaped to torpedo-shaped embryos. In the wild type and mutants, the SEL of heart-shaped embryos allowed for 10-kDa FITC-dextran to move through the cells; the SEL was significantly decreased in torpedo-shaped embryos, and the 10-kDa FITC-dextran no longer moved through plasmodesmata. By contrast, both *ise1* and *ie2* mutants still maintained the SEL and allowed 10-kDa FITC-dextran to move into adjacent cells in torpedo-shaped embryos (Kim et al. 2002). Thus, increased SEL may allow movement of larger macromolecules. Our soybean endosperm cells allowed movement of at least 40-kDa FITC-dextran, so the SEL of plasmodesmata in soybean endosperm cells was significantly larger than that in Arabidopsis embryo cells and soybean cotyledon epidermis cells. Thus the transport speed of tracers in soybean endosperm was faster than Arabidopsis seed and soybean cotyledon epidermis.

The weight of legume seeds varies widely, from 0.1 mg (e.g., *Trifolium* spp.) (Zoric et al. 2010) to > 1000 g (e.g., *Mora oleifera*) (Boesewinkel and Bouman 1984). Some legume endosperm show a positive relation between the presence of nitrogen-fixing nodules and the size of endosperm (Corby et al. 2011), although this is not the case for most legume crops. For example, the weight of 100 seeds (g/100 seeds) of *M. truncatula*, pea, soybean, and faba bean is 0.3, 12.5, 20, and > 50, respectively. Two of these four species, pea and faba bean, have no endosperm, but seeds of pea and faba beans contain two layers of transfer cells (TCs), one layer differentiated from parenchyma of maternal seed coat and the other layer differentiated from epidermis of fetal cotyledons (Thompson et al. 2001). Several studies demonstrated that these TCs have important roles in solute transport from source to sink (Offler and Patrick 1993; Patrick et al. 1995; McDonald et al. 1996; Tegeder et al. 1999; Patrick and Offler 2001; Rosche et al. 2002). In cereal kernels, endosperm TCs (ETCs) differentiate from endosperm cells facing the major vascular bundle (Brown and Lemmon 2007). ETC-related mutants also demonstrated that defective ETCs caused small and abnormal kernels, which suggests that ETCs are a mediator of solute flow from maternal TCs in the vascular bundle to starchy endosperm cells (Miller and Chourey 1992; Cheng et al. 1996; Gomez et al. 2009; Royo et al. 2007). However, based on current ultrastructural observation of soybean seeds, there is no significant evidence to show that the parenchyma of the soybean seed coat or cotyledon epidermis have visible TCs (Thorne 1981; Yaklich et al. 1984; Chamberlin et al. 1994). According to current knowledge, many seeds, including those of Arabidopsis and *M. truncatula*, have no visible TCs in seed tissues. In Arabidopsis, nutrient flow involves a complex set of cell layers and tissues in the integument and epidermis in embryos (Stadler et al. 2005a; Chen et al. 2015). Considering the extreme difference in seed size between soybean and Arabidopsis (200 vs 0.02 g/1000 seeds), a specialized tissue may be important to improve transport efficiency of nutrient flow.

In the current study, we illustrate that soybean endosperm tissue covers the cotyledon and embryo, and the living layer is fully alive at mid-developmental stage, a period with highly accumulating nutrient transport into sink tissues (e.g., cotyledon). From the results of cell-to-cell movement assays, we provide evidence that soybean endosperm may enhance the transport speed and amount of available macromolecules. Considering the poor transport ability on the cotyledon surface shown in this work, soybean endosperm may play a role of mediator of moderate nutrient flow released from the seed coat because of its characteristics of fast horizontal transport.

## Data Availability

Not applicable.

## Abbreviations

DAF: days after flowering
DCCD: *N*,*N*′-dicyclohexylcarbodiimide
ETC: endosperm transfer cell
FITC: fluorescein isothiocyanate
GFP: green fluorescent protein
HPTS: 8-hydroxypyrene-1,3,6-trisulfonic acid
MS: Murashige and Skoog
PI: propidium iodide
SEL: size exclusion limit
SEM: scanning electron microscopy
TC: transfer cell
